# Turning the Cocopith Waste into Myceliated Biocomposite to Make an Insulator

**DOI:** 10.1155/2021/6630657

**Published:** 2021-05-18

**Authors:** Diana Susyari Mardijanti, Erri Noviar Megantara, Ayi Bahtiar, Sunardi Sunardi

**Affiliations:** ^1^Graduate Program on Environmental Studies, Postgraduate School, Universitas Padjadjaran, Bandung, Indonesia; ^2^Department of Biology, Faculty of Mathematics and Natural Science, Universitas Padjadjaran, Sumedang, Indonesia; ^3^Department of Physics, Faculty of Mathematics and Natural Science, Universitas Padjadjaran, Sumedang, Indonesia; ^4^Center for Environment and Sustainability Science, Universitas Padjadjaran, Bandung, Indonesia

## Abstract

Cocopith is the main waste of the coconut coir milling industry, which has not been handled properly until now. Burning cocopith as a response to land availability concerns for storing waste has an impact on pollution for the surrounding environment. Efforts to reduce, reuse, recycle, and remanufacture cocopith waste provide better economic value for waste. The method used in this research is one with quantitative and qualitative approaches. The AAS method is used to test the concentration of cocopith chemical elements, while lignin and cellulose levels were tested using data methods. The test results obtained that the highest chemical elements are sulfur and chlorine; the sulfur content in 1 kg of cocopith is 24,000 mg and chlorine content is 10,371 mg. Meanwhile, the other results showed that lignin levels in cocopith (22.7%) are higher than cellulose content (10.27%). The test results of cocopith characteristics from the methods mentioned above showed that the chemical content of sulfur and chlorine and lignin, more so than cellulose, causes cocopith to have the potential to insulate thermally. Based on this potential, cocopith is processed into mycelium-based biocomposite that serves as an insulator. Maximum stress and tensile stress of this biocomposite have been tested through flexural strength tests with the ASTM-D7264 method. The biocomposite feasibility of the material as an insulator was shown through a thermal conductivity test at temperatures of 13°C–40°C. This showed a thermal conductivity value of 0.0887241 ± 0.002964 W/mK. This value is in the range of 0.01–1.00 W/mK, which is a recommended value for the thermal conductivity insulator.

## 1. Introduction

Indonesia, as the world's largest coconut producing country, is estimated to produce 14 billion coconuts each year [1]. Coconut commodities became the country's mainstay export because it brings many benefits to the country in general and local communities in particular. Nevertheless, coconut commodity processing has not been maximal because processing is only done to obtain fresh coconut or copra, the raw material of coconut oil production. Meanwhile, the by-product of the treatment, the coconut fiber, is still underutilized and considered only as waste [2].

Each grain of coconut contains about 65% kernel weight (the shell, fruit meat, and water) and 35% coconut fiber (*husk*). Coconut fiber (*husk*) consists of 70% *pith* (coconut fiber powder) and 30% coconut fiber (*coir fiber*) per dry weight base [3]. Based on this ratio, it will certainly cause new problems if there are no coconut fiber waste treatment plans. Cocopith, which is a network of pith or often called “cork,” is the part that connects the fiber strands to each other and becomes the main waste of the coconut coir processing industry, which until now has still not been handled properly. This waste is left dumped openly near the coconut fiber industry and may contaminate the environment.

Uncontrolled and increasing deposits of cocopith waste will lead to the transformation of productive land into wasteland. The wastewater from the landfill contains chemical elements of cocopith, which in a large percentage interferes with the water habitat around the landfill. The practical step taken by the community to reduce this landfill was to burn the material at the local site. However, burning is not the right solution as it adds to the problem of air pollution. As reported by D. D. A. Ravindranath [4], despite having the potential to be a fuel with an energy value almost equivalent to coal (3975 kcal/kg), imperfect combustion properties lead to the release of very large amounts of greenhouse gases into the air, including sulfur dioxide (SO_2_), nitrogen oxide (NO_2_), and carbon monoxide (CO) gases and particulates, which are extremely detrimental to public health around the mill site.

The problem of cocopith deposits is a major problem, as the coconut coiling industry continues to produce to meet market demand. The treatment of cocopith waste must be carried out immediately so that existing problems can be resolved and so the economic value of waste can be increased. The use of coconut fiber powder as a composite board material for furniture and acoustic purposes—among them the manufacture of composite boards with polyethylene mixtures [5], polyester resins [6], and coconut cograin powder composites with a Styrofoam [7]—is an active research topic to date. In addition, cocopith is also utilized in the manufacture of biobriquettes with *a mixture of bottom ash* [8] as an artificial planting medium and coconut fiber net *(cocomesh)* for the reclamation of former mining land [2] and as a coconut coir biomass filter [9].

Due to economic reasons or practicality, existing methods of utilization have not been able to properly solve this problem. Therefore, it is necessary to seek alternatives to other utilization methods that are more practical and economical in the sources of the waste arising. The utilization of this waste should embody circular economic principles, namely, economic systems based on the principle of reducing, reusing, or recycling and restoring materials in the production and distribution process for later consumption by people (*reduce, reuse, recycle, and remanufacture*). Additionally, solutions should provide better economic value to waste and residues and add benefits through efficiency in raw materials and production processes while ensuring a clean environment and economic prosperity for current and future generations [10].

Waste treatment of myceliated cocopith biocomposite for insulator products is very prospective and is an environmentally friendly process. This is because the composite formation process does not require chemicals, but cocopith is used as a substrate of wood fungus (*Ganoderma lucidum* lingzhi), which is maintained by the growth conditions so that the roots of the fungus (mycelium) will bind to each cavity between the particles of cocopith and become a solid or novel material. This research aims to examine the material characteristics *of cocopith* and evaluate whether this mycelium-based biocomposite is worthy of being an insulator.

The function of the insulator as a heat retainer will contribute to various aspects of life. The use of coconut fiber thermal panels as wall insulation in people's homes in Nepal provides energy savings due to the reduction in carbon emissions resulting from burning wood in the house for heating. Energy savings during winter in Bhuj are around 6%–11% in a year which is equivalent to 964 kwh [11].

The biocomposite that has been developed for sustainable construction and also functions as an insulator is Thermacork. The panels made from the outer bark of the oak tree have very low thermal conductivity and high hygrothermal efficiency. The development of this biocomposite uses nanotechnology to increase water resistance and protect against biodegradation [12].

## 2. Materials and Methods

Production *of myceliated biocomposite as* a treatment of cocopith waste with the following stages.

### 2.1. Raw Materials

The raw material of the manufacture of biocomposite is cocopith obtained from the remaining milled coconut fibers at one of the milling locations in Parigi, Pangandaran.

### 2.2. Biocomposite Manufacturing Procedure

#### 2.2.1. Cocopith Biological Test

The thermal potential of cocopith becomes clear when considering its content of lignin and cellulose, which are the main components of plant cell wall constituents. The test of lignin and cellulose levels in cocopith was conducted using the data method put forward by Chesson [13].

#### 2.2.2. Cocopith Chemical Test

The chemical composition contained in cocopith affects its thermal properties. The chemical content test of cocopith against elements of calcium, sodium, aluminum, chlorine, potassium, and sulfur was carried out using the AAS method (*Atomic Absorption Spectrophotometry*).

#### 2.2.3. Myceliated Biocomposite Manufacturing Process

The process of making biocomposite is done at MYCL (*Mycotech Lab*), Bandung. The process of making biocomposite is as follows:

- Cocopith that is already dry (water content reduced by 50%) mixed with wood powder, 15% pollard (bran), 3% lime, 5% tapioca, and ganoderma mushroom seeds and then put into baglogs.
- Each baglog contains 500 grams of a mixture of cocopith composition and wood powder mixed to different degrees until mycelium will grow well and form solids.
- Baglog is set by using an autoclave pressurized at 0.15 MPa, which is conditioned at a temperature of 126°C–130°C for 1 hour.
- The baglog is incubated at a temperature of 22°C-23°C to maintain the optimal environment state where mycelium will grow on this substrate of cocopith.
- The baglogs that are overgrown with mycelium on the 10^th^ day are transferred into a mold, the size of which is adjusted to the research needs and left until mycelium grows throughout the entire baglogs.
- The baglogs that are solid are then removed from the molds and dried until the water content is reduced by 10%. Compaction is done by hot press process so that solid biocomposite is formed according to the expected size.

### 2.3. Biocomposite Characteristics Test

The biocomposite produced is expected to have good mechanical capabilities as particle board with potential multifunction so that it can play a role in the industry. The physical properties of biocomposite are tested for *maximum stress* and *tensile stress*, using the compressive test method and tensile test (flexural strength test using the ASTM-D7264 method) in the laboratory.

### 2.4. Biocomposite Feasibility Evaluation

The feasibility of biocomposite as an insulator can be shown through thermal conductivity tests. The cocopith biocomposite was transformed into insulation material by taking parameters of thermal conductivity values resulting from such biocomposite. The testing device used is a *guarded hot-plates* system operated with reference to *ASTM C177-04* [14]. Testing is carried out by measuring temperature and electrical power to determine the rate at which the calorific flow penetrates the test object and the temperature difference between the surfaces of the test object.

## 3. Results and Discussion

The cocopith and biocomposite cocopith test provides results as follows.

### 3.1. Cocopith Biological Test

Cocopith is a biological waste that is a biomass lignocellulose whose main content is cellulose and lignin. Cellulose and lignin test results are found in Table 1.

From Table 1, lignin content in cocopith (22.70%) is greater than cellulose (10.27%). Lignin is the most powerful material in biomass, but it is very difficult to degrade. It has thermoplastic properties that can soften at high temperatures (120°C) [15]. Lignin is a highly effective and economical adhesive material, acting as a compatible binding material for the manufacture of biocomposite [16]. The properties of cellulose and lignin may explain the characteristics of hard-to-decompose cocopith and the thermoplastic properties of lignin that can be analogous to resin properties that have good insulation capabilities.

### 3.2. Cocopith Chemical Test

Chemical concentration test results in cocopith, analysis no. S-006/LS-AK.29/2019, are described in Table 2.

From Table 2, based on the test results of cocopith constituents, a comparison was made of the composition of elements Ca, Na, Al, Cl, K, and S. In the 1 kg of cocopith tested, there was a sulfur content of 24,000 mg. Sulfur comprises the greatest content of cocopith, compared to other chemical compounds. In addition, sulfur can be used as thermal insulation [17]. This is due to the low sulfur thermal conductivity of 0.268 W/mK at an average temperature of 33°C [18].

The chlorine content in cocopith is highest after sulfur. In 1 kg of cocopith there is 10,371 mg of chlorine. The ability of chlorine to drain heat in each unit of length (meters) for every 1 Kelvin is 0.0089 Watts, meaning that only a small amount of heat can be produced by chlorine compounds. This is even less heat that can be produced by chlorine or sulfur. Based on the thermal conductivity of both elements, chlorine and sulfur will withstand heat from moving to the other side. It can be concluded that sulfur and chlorine have the potential to insulate well and, thus, to be insulators.

### 3.3. Mycelium Growth Results in Baglog to Produce Biocomposite

The percentage of the composition of cocopith and different wood powders causes the growth of different fungi in the baglog. The growth of mycelium in baglogs occurs differently in the composition of cocopith and a piece of wood. Cocopith and wood powder have become the main raw materials of this biocomposite. In addition, it is also necessary to use other materials, as much as 15% pollard (bran), 3% lime, and 5% tapioca, to aid the growth of mycelium.

The mycelium that grows on this substrate is a vegetative part of the fungus in the form of white filament-shaped tissue. Mycelium acts as a natural *adhesive* so that the formed composites will be free of *formaldehyde and other additive adhesives.* As a binding material, the mycelium will grow over a certain period to form a strong and dense structure into a composite and can also grow into a strong and flexible skin-like material. This product aims not only to achieve *biodegradable product* targets but also to achieve the target of utilizing biological waste into environmentally friendly consumer products with low production costs. Due to this efficiency, it has great potential as a sustainable biomaterial of the future [19].

Here are the observations of the growth of mycelium on the composition of cocopith and different wood grains (Table 3).

From Table 3, the composition of cocopith (50%) and wood powder (27%) provides the best results for mycelium growth in the baglog. The physical differences in biocomposite for each different composition of cocopith and wood powder are explained in Table 4.

From Table 4, in composition D, with cocopith (50%) and wood powder (27%), it appears that mycelia grow thick and evenly throughout the substrate and can be removed from the mold, resulting in solids. The water content in the solids should be reduced to 10% from the initial state by heating at 110°C so that mycelium does not grow again [20]. Compaction finalization is done with a *hot press* at a pressure of 10 tons to become biocomposite.

### 3.4. Biocomposite Characteristics as Insulator

#### 3.4.1. Biocomposite Mechanical Characteristics

The following data were obtained from test results for bending the voltage value (*maximum flexural stress*) with the ASTM-D7264 method in the laboratory (Table 5):

From Table 5, biocomposite produced from a 500-gram mass baglog consisting of a mixture of cocopith, wood powder, and other material mixtures have a pressing strength ranging from 7.67 MPa–7.97 MPa to a thickness of 6.67 mm–6.73 mm, which explains the voltage's ability to deform a particle board or the ability of the particle board to withstand shape changes. This pressing strength value can be increased by increasing the number of baglogs to one required size. The more baglogs are put together into a single volume size, the denser are the resulting biocomposite and the greater the value of biocomposite press strength.

The test results for *tensile strength* with the ASTM-D7264 method in the laboratory are explained in Table 6:

From Table 6, biocomposite tensile strength *increases elongation* of 0.27%–0.32% of the initial state when it obtains a tensile force on the particle board. The strength of the particle board depends on the homogeneity of particle board geometry—the more homogeneous the particle board, the fewer air cavities [21]. The homogeneity of this biocomposite relies heavily on the spread of mycelium growth in the substrate, so the initial process of the formation of this biocomposite should be carefully considered especially in terms of the composition of raw materials used. The formation of biocomposite is also strongly influenced by mycelium containing vitamin and mineral enzymes that grow well on the substrate by the 15^th^ day [22].

#### 3.4.2. Biocomposite Feasibility Evaluation

Biocomposite thermal conductivity testing using a testing device in the form of a *guarded hot-plates* system is operated by referring to *ASTM C177-04* [11]. Thermal conductivity is calculated as follows:

(1) $$\begin{array}{c} \lambda =\frac{qL}{A\left(T_{h}-T_{c}\right)}, \end{array}$$

here, *A* = specimen area normal to heat flux direction, m^2^, *λ* = thermal conductivity or apparent thermal conductivity, W/(mK), *T*_*h*_ = area-weighted temperature of specimen hot surface, K, *T*_*c*_ = area-weighted temperature of the specimen cold surface, K, *L* = specimen thickness, m, and *q* = heat flux (heat flow rate per unit area), *Q*, through area, *A*, W/m^2^.

Biocomposite thermal conductivity test results at temperature (13–40°C) have a thermal conductivity value of 0.0887241 ± 0.002964 W/mK. The thermal conductivity value is still in the range of 0.01–1.00 W/mK as a recommendation of the thermal conductivity value of the insulator [23]. This results in the physical condition of this biocomposite being harsh, dense, and nonflexible. When exposed to heat, it does not undergo deformation and also does not cause odors, which can indicate the absence of the release of a chemical compound.

## 4. Conclusions

Cocopith as residue from the coconut coir milling industry has the greatest number of chemical compounds, namely, sulfur and chlorine. Both chemical compounds play a role in holding in heat through the medium of cocopith. This insulating characteristic is also reinforced by more lignin content than cellulose possessed by cocopith. Lignin, which has thermoplastic properties, can be considered a resin that has low thermal conductivity so that it can be utilized to insulate thermals that pass through certain mediums.

This biocomposite produced with cocopith-based material has a thermal conductivity value of 0.0887241 ± 0.002964 W/mK and can be recommended as a biocomposite that serves as an insulator. The utilization of insulators as thermal restraints can be used in various sectors, such as the food industry and the housing, electrical, and machinery industries. Any equipment that uses machines requires heat exchange in order for the machine to function properly.

The advantages of the cocopith biocomposite as an insulator are that these biocomposite characteristics can be mechanically conditioned according to the needs of the industry, based on tensile strength and pressure test (maximum flexural stress), where strong press value and strong biocomposite pull is directly proportional to the density value of the biocomposite itself. When compared to other insulators, the thermal conductivity values of wood grain insulators are 0.05 W/mK. Styrofoam (0.3 W/mK) and polyurethane are 0.025 W/mK. Cocopith raw material insulators have high water content (119%) or have a high water shelf life (695%) [22], which means that it has the ability to absorb liquids so that it is not easily destroyed and has a longer life span than other insulators.

## Figures and Tables

**Table 1:** The results of the lignocellulose composition test on cocopith.

Materials	Composition	Content (%)
Cocopith	Cellulose	10.27
	Lignin	22.70

Analysis no. S-068/LS-AK.30/2019.

**Table 2:** Chemical test results in cocopith with AAS-Unpad method.

Concentrate (mg/kg)					
Ca	Na	Al	Cl	K	S

719	6,450	592	10,371	11,000	24,000

**Table 3:** Mycelium growth in baglog.

No.	Material composition (%)		Mycelium growth (%)	Subject composition
	Cocopith	Wood powder		
1	77	0	0	
2	70	7	0	
3	65	12	17.72	A
4	60	17	36.81	B
5	55	22	76.74	C
6	50	27	87.71	D

**Table 4:** Percentage of mycelium growth ratio in baglogs.

Photos during observation	Total amount (pixel)	Total of growing fungi (pixel)	Percentage of growing fungi (%)
Composition A 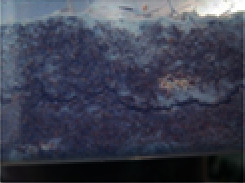	14,000 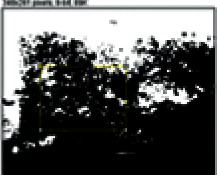	2481	17.72
Composition B 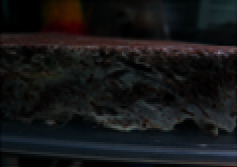	16,900 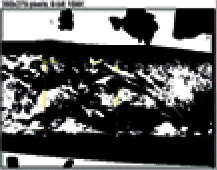	6221	36.81
Composition C 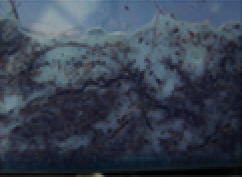	14,000 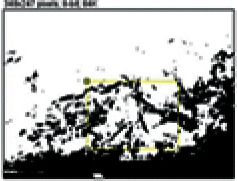	10,743	76.74
Composition D 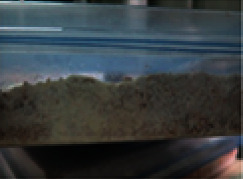	9660 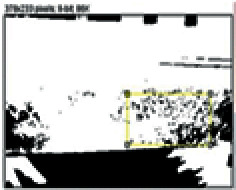	8473	87.71

**Table 5:** Biocomposite press strength.

No.	Specimens	Thickness (mm)	Width (mm)	Area (mm^2^)	Max load (N)	Maximum flexural stress (MPa)
1	Biocomposite 1	6.67	17.93	119.59	29.13	7.67
2	Biocomposite 2	6.71	17.96	120.51	30.78	7.99
3	Biocomposite 3	6.73	18.23	122.69	31.23	7.94

**Table 6:** Biocomposite *tensile strength*.

No.	Specimens	Thickness (mm)	Area (mm^2^)	Max load (N)	Extension (mm)	Tensile strength (MPa)	Elongation (%)
1	Biocomposite 1	4.35	43.94	5.25	0.11	0.12	0.27
2	Biocomposite 2	4.00	40.00	4.14	0.14	0.1	0.32
3	Biocomposite 3	3.9	37.44	2.15	0.11	0.57	0.27

## Data Availability

The raw material of cocopith was obtained from the coconut fibers milling in Parigi, Pangandaran, Indonesia.

## References

[1] Widananto H., Purnomo H. (2013). Rancangan mesin pengupas sabut kelapa. *Jurnal Teknik Mesin Institut Teknologi Padang*.

[2] Oktavia F. (2013). Peran produk olahan sabut kelapa sebagai penunjang kelestarian ekologi. *Prosiding*.

[3] Kimia M. T., Wildan A. (2010). Studi proses pemutihan serat kelapa program pascasarjana universitas diponegoro.

[4] Ravindranath D. D. A. (2016). Coir pith wealth from waste a reference.

[5] Prasetyawan D. (2009). Sifat fisis dan mekanis papan komposit dari serbuk sabut kelapa (cocopeat) dengan plastic polyethylene.

[6] Djiwo I. S., Sugiarto T., Setyawan E. Y. (2016). Pemanfaatan gabus sabut kelapa (cocopeat) sebagai komposit yang berbasis ramah lingkungan. *Jurnal Flywheel*.

[7] Nurhajati D. W., Indrajati I. N. (2011). Kualitas komposit serbuk sabut kelapa dengan matrik sampah. *International Journal of Research in Industrial Engineering*.

[8] Sinta Rismayani A. S. T. B. (2011). Pembuatan bio-briket.

[9] Pinandari A. W., Fitriana D. N., Nugraha A., Suhartono E. (2011). Uji Efektifitas dan efisiensi filter biomassa menggunakan sabut kelapa (Cocos Nucifera) Sebagai bioremoval untuk menurunkan kadar logam (Cd, Fe, Cu), Total padatan tersuspensi (TSS) dan meningkatkan pH pada limbah air asam tambang batubara. *Journal Prestasi*.

[10] Kirchherr J., Reike D., Hekkert M. (2017). Conceptualizing the circular economy: an analysis of 114 definitions. *Resources, Conservation and Recycling*.

[11] Brose A., Kongoletos J., Glicksman L. (2019). Coconut fiber cement panels as wall insulation and structural diaphragm. *Frontiers in Energy Research*.

[12] Ansell Y. J. H. M. H. L. (2020). Physico-chemical characterization and development of hemp aggregates for highly insulating construction building materials. *Sustainable Agriculture Reviews*.

[13] Mudyantini W. (2008). Pertumbuhan, Kandungan Selulosa, dan Lignin pada Rami (Boehmeria nivea L. Gaudich) dengan Pemberian Asam Giberelat (GA3). *Biodiversity*.

[14] ASTM International (2021). Standard test method for flexural properties of polymer matrix composite Materials. *Standard Test Method for Flexural Properties of Polymer Matrix Composite Materials*.

[15] Brebu M., Vasile C. (2010). Thermal degradation of lignin-a review. *Cellulose Chemistry and Technology*.

[16] Polat K. A., Stojanovska E., Negawo T. A., Doner E. (2017). *Lignin as an Additive for Advanced Composites*.

[17] Abraham A. M., Kumar S. V., Alhassan S. M. (2017). Porous sulphur copolymer for gas-phase mercury removal and thermal insulation. *Chemical Engineering Journal*.

[18] Sugawara A. (1965). Thermal conductivity of sulfur accompanying crystal transition and phase change. *Journal of Applied Physics*.

[19] Joey Yang Z., Zhang F., Still B., Amstislavski P. (2017). Physical and mechanical properties of fungal mycelium-based biofoam. *Journal of Materials in Civil Engineering*.

[20] Jiang L., Walczyk D., Mcintyre G. A new process for manufacturing biocomposite laminate and sandwich parts using mycelium as a binder.

[21] Rahmi M. (2019). pengaruh variasi panjang serat ampas tebu dan serbuk kulit buah kakao terhadap sifat fisis. *Mekanis, Dan Konduktivitas Termal Papan Partikel*.

[22] Parjimo H., Soenanto D. (2008). Jamur lingzhi raja herbal, seribu khasiat.

[23] Bergman T. L. (1966). *Introduction to Heat Transfer*.

